# Discrete Emotion Profiles in Old Age: Stability and Change for Better or Worse

**DOI:** 10.1093/geroni/igab046.2200

**Published:** 2021-12-17

**Authors:** Jeremy Hamm, Carsten Wrosch, Meaghan Barlow, Ute Kunzmann

**Affiliations:** 1 North Dakota State University, Fargo, North Dakota, United States; 2 Concordia University, Montreal, Quebec, Canada; 3 University of California, Berkeley, University of California, Berkeley, California, United States; 4 Leipzig University, Leipzig, Sachsen, Germany

## Abstract

Although discrete emotions can change in salience across adulthood, little is known about developmental shifts in the co-occurrence of multiple discrete emotions. The present study (n=389, Mage=73) adopted a person-centered approach to identify stability and change in commonly-occurring profiles of calmness, excitement, sadness, and anger. Daily emotions were assessed over 1-week periods at baseline and two years later. Latent class analyses yielded consistent 3-profile solutions at both waves: a positive emotion (high calmness-moderate excitement-low sadness and anger), a mixed emotion (moderate/high calmness-moderate excitement, sadness, and anger), and an apathetic emotion profile (low calmness, excitement, sadness, and anger). Latent transition analyses revealed both stability (82% remained in the same profile) and change (18% changed profiles) in profile membership. Higher baseline optimism and fewer chronic conditions were associated with adaptive changes in profile membership. Findings point to the importance of considering the co-occurrence of distinct emotions in studying emotional aging.

